# First Quantification of Calcium Intake from Calcium-Dense Dairy Products in Dutch Fracture Patients (The Delft Cohort Study)

**DOI:** 10.3390/nu6062404

**Published:** 2014-06-23

**Authors:** Peter van den Berg, Paul M. M. van Haard, Joop P. W. van den Bergh, Dieu Donné Niesten, Maarten van der Elst, Dave H. Schweitzer

**Affiliations:** 1 Department of Orthopedics and Surgery, Reinier de Graaf Group of Hospitals, 2625AD Delft, The Netherlands; E-Mail: pberg@rdgg.nl; 2 Department of Medical Laboratories/Diagnostic Centre SSDZ, Reinier de Graaf Group of Hospitals, Association of Clinical Chemistry, 2625AD Delft, The Netherlands; E-Mail: haard@rdgg.nl; 3 Department of Internal Medicine, VieCuri Medical Centre Noord-Limburg 5912 BL Venlo, The Netherlands; E-Mail: j@vdbergh.org; 4 Department of Internal Medicine, Subdivision Rheumatology, Maastricht University Medical Centre, 6229 HX Maastricht, The Netherlands; 5 Department of Orthopaedics, Reinier de Graaf Group of Hospitals, 2625AD Delft, The Netherlands; E-Mail: niesten@rdgg.nl; 6 Department of Surgery, Reinier de Graaf Group of Hospitals, 2625AD Delft, The Netherlands; E-Mail: elst@rdgg.nl; 7 Department of Internal Medicine, Reinier de Graaf Group of Hospitals, Reinier de Graafweg 3–11, 2625AD Delft, The Netherlands

**Keywords:** calcium-dense food, dairy products, Fracture Liaison Service, FRAX

## Abstract

Recommendations for daily calcium intake from dairy products are variable and based on local consensus. To investigate whether patients with a recent fracture complied with these recommendations, we quantified the daily dairy calcium intake including milk, milk drinks, pudding, yoghurt, and cheese in a Dutch cohort of fracture patients and compared outcomes with recent data of a healthy U.S. cohort (80% Caucasians). An observational study analyzed dairy calcium intakes of 1526 female and 372 male Dutch fracture patients older than 50. On average, participants reported three dairy servings per day, independently of age, gender or population density. Median calcium intake from dairy was 790 mg/day in females and males. Based on dairy products alone, 11.3% of women and 14.2% of men complied with Dutch recommendations for calcium intake (adults ≤ 70 years: 1100 mg/day and >70 years: 1200 mg/day). After including 450 mg calcium from basic nutrition, compliance raised to 60.5% and 59.1%, respectively, compared to 53.2% in the U.S. cohort. Daily dairy calcium intake is not associated with femoral neck bone mineral density (BMD) T-scores or WHO Fracture Assessment Tool (FRAX) risk scores for major fracture or hip fracture. However, when sub analyzing the male cohort, these associations were weakly negative. The prevalence of maternal hip fracture was a factor for current fracture risks, both in women and men. While daily dairy calcium intake of Dutch fracture patients was well below the recommended dietary intake, it was comparable to intakes in a healthy U.S. cohort. This questions recommendations for adding more additional dairy products to preserve adult skeletal health, particularly when sufficient additional calcium is derived from adequate non-dairy nutrition.

## 1. Introduction

In 1993, the U.S. Food and Drug Administration authorized a health claim for foods and supplements related to calcium to prevent osteoporosis. In January 2010, the health claim was expanded for the combination of calcium and vitamin D supplements. (FDA 21CFR101.72) This claim raises questions about the amount of calcium that should be recommended to the adult population [1]. Today, the Recommended Dietary Intake (RDI) for calcium, meaning the average daily level of intake sufficient to meet the nutrient requirements of healthy individuals, is 1200 mg for individuals older than 50 years in the U.S. (Food Guide Pyramid FGP, U.S. Department of Agriculture) [2]. In the Netherlands, the RDI for calcium is quite similar to that in the U.S.: 1100 mg for the age category 51–69 years and 1200 mg for people older than 70 years. Based on the US recommendation, the number of recommendable calcium-dense servings should be four per day [2,3], which is more than the actual average consumption in the Netherlands as well as in the USA. While extra intake of dairy servings would seem the most straightforward way to increase calcium intake, it is questionable whether it is beneficial for bone health to use more dairy servings than those taken according to cultural habits and traditions. Overnutrition of dairy products may cause maldigestion and malabsorption, which may overrule beneficial effects of calcium intake. Besides, it is unclear how traditional use of liquid milk and milk products relates to bone mineral density (BMD) and fracture risk during adulthood.

Public health authorities [4] claim that actual calcium intake is 25 percent lower in Europe than in the U.S. Although a large part of the agricultural activities in the Netherlands is dedicated to milk production and processing, the average Dutch consumption of liquid milk and milk products *per capita* in kg/year is estimated to be 83.5, while it is 85.1 on average in 27 other European countries and 111.4 in the U.S. [5]. According to the Dutch National Food Consumption Survey (DNFCS 2007–2010), the median habitual total calcium intake per day (51–69 years; P50) in Dutch men is 1109 mg calcium and in women 985 mg [6]. Non-dairy food sources contribute 42% of total calcium content in daily nutrition [6].

Since dairy product consumption *per capita* in kg/year in the Netherlands compares well with the average in North-Western Europe, we were interested in comparing calcium intake in the Dutch adult population with that of an U.S. cohort of more than 80% Caucasians, which was extracted from the Continuing Survey of Food Intake by Individuals, 1994–1996, 1998, (CSFII), and the National Health and Nutrition Examination Survey, 1999–2000 (NHANES). We wondered whether the CSFII and NHANES reports would agree with the number of daily servings consumed by Dutch individuals.

In order to answer this question, we conducted the present study at the Fracture Liaison Service (FLS) of the Reinier de Graaf Group of Hospitals in The Netherlands. Our primary research goals were to determine the number of daily servings of dairy products in a Dutch cohort of fracture patients after partitioning for age and gender, but also for population density; to correlate daily calcium intake from dairy products with femoral neck BMD T-scores and 10-year probability of major fracture or hip fracture; and to compare intakes with another, mainly Caucasian cohort in the U.S., consisting of patients without reported fractures. We further question whether the total amount of calcium from both daily basic nutrition and daily dairy consumption is appropriate for fulfilling Dutch recommendations for total calcium intake.

## 2. Experimental Section

### Patients and Methods

This prospective observational study was conducted at the Fracture Liaison Service (FLS) of the Reinier the Graaf Group of Hospitals, Delft (The Netherlands). The FLS is organized according to a previously reported concept [7,8,9]. We included women and men of 50 years of age and older with a recent fracture. These patients were evaluated by means of a structured diagnostic work up, including a detailed questionnaire regarding their daily calcium intake from dairy and a Dual Energy X-ray Absorptiometry (DXA) measurement for femoral neck BMD T-scores. Each patient received a scan, with the exception of those who were already known with osteoporosis evidenced by older DXA measurements or with prevalent vertebral fractures evidenced by previous spinal radiographs. Patients using prescribed calcium and/or bisphosphonates were excluded.

Calcium intake was assessed using a validated Dutch questionnaire on Daily Calcium Intake (DCI-Holland), which was developed by Het Rijksinstituut voor Volksgezondheid en Milieu (National Institute for Public Health and the Environment, RIVM), Bilthoven, the Netherlands [4]. The DCI-Holland calculates the calcium content of food categories such as meat, fish, poultry, eggs, vegetables, fruits, potatoes, pasta, and milk products. According to the DCI-Holland, the average calcium content is 270 mg in one glass of milk (200 mL), 400 mg in one glass of calcium-enriched milk, and 240 mg in one serving of yoghurt or pudding (200 mL). Non-dairy food contributes (as median) 430 mg calcium in females and 443 mg calcium in males, based on the habitual intake distribution at P50 of calcium food sources by the Dutch population aged 51–69 years, weighted for socio-demographic factors, season, and day of the week.

Patients were recruited from the Emergency Department or were outpatients of the departments of trauma surgery or orthopedic surgery. Each patient was asked to fill in a questionnaire that included questions about calcium intake from dairy nutrition categorized per product type (milk, calcium-enriched milk, yoghurt, pudding, cheese) as mentioned in the DCI-Holland. We estimated total daily dairy calcium intake using the number of glasses of milk or yoghurt/pudding servings. The calcium intake of cheese products was dichotomized (Yes/No). The intake of calcium from cheese products was calculated as half the amount in a glass of milk. The questionnaire also included the WHO Fracture Assessment Tool (FRAX) for evaluating fracture risks [10] and additional questions regarding age at menopause, daily exercise, and sports habits. Calcium intake from dairy nutrition per serving was calculated using the formula of Fulgoni [2], with one serving representing 250 mg of calcium according to the Dutch Standard (DCI-Holland).

FRAX risk scores were calculated according to the Dutch FRAX algorithm [11]. Osteoporosis was defined as a T-score ≤ −2.5 SD (SD = standard deviation) at either the total hip, femoral neck or lumbar spine, normal BMD as a T-score ≥ −1 SD at all three locations and osteopenia as a T-score between −1 SD and −2.5 SD.

In patients with a T-score of less than −2 SD, additional laboratory assessments and radiographs of the lumbar and thoracic spine were performed according to protocol. Vertebral fractures were scored according to the semi-quantitative method of Genant *et al.* [12]. A vertebral fracture was defined as an infraction of at least 25% reduction in anterior, middle, and/or posterior height and a reduction in area by 20%–40% [12].

Analyses were performed on five age decades (50–59, 60–69, 70–79, 80–89 and 90–100 years), gender, and on five (Dutch) population density categories (1:21–250, 2:250–500, 3:500–1000, 4:1000–2500 and 5:2500–5976 inhabitants per square kilometers according to the Dutch National Atlas of Public Health [13].

Data were analyzed using Statgraphics Centurion XVI software (Version 16.2.4 for MS-Windows; Statpoint, Inc., Warrenton, VA, USA). Multiple-variable correlation analysis was applied after partitioning of data by gender. Wilcoxon & Mann & Whitney rank-sum test was applied to compare medians of numerical variables with respect to gender. Mood’s Median and Kruskall & Wallis tests were applied to genders in order to compare numerical variables between the categories of the diagnosis variable (normal, osteopenia, osteoporosis). Variance Components Analysis was applied, using fracture frequency as a dependent variable and relevant numerical variables as factors in order to assess their contribution to total variation in fracture frequency. To estimate odds ratios in genders, logistic regression with backward factor selection (p-to-enter 0.05; p-to-remove 0.05) was applied, with maternal hip fracture as dependent variable (Yes = 1; No = 0) and all numerical and dichotomous categorical variables as factors. Non-parametric correlations were then assessed again between final numerical factors of the best logistic regression model. Where applicable, 95% confidence intervals were used and a *p*-value < 0.05 was considered statistically significant at the 95% confidence level. The study was approved by the Medical Ethical Review Board (METC Zuidwest Holland), The Netherlands.

## 3. Results

**Figure 1:**
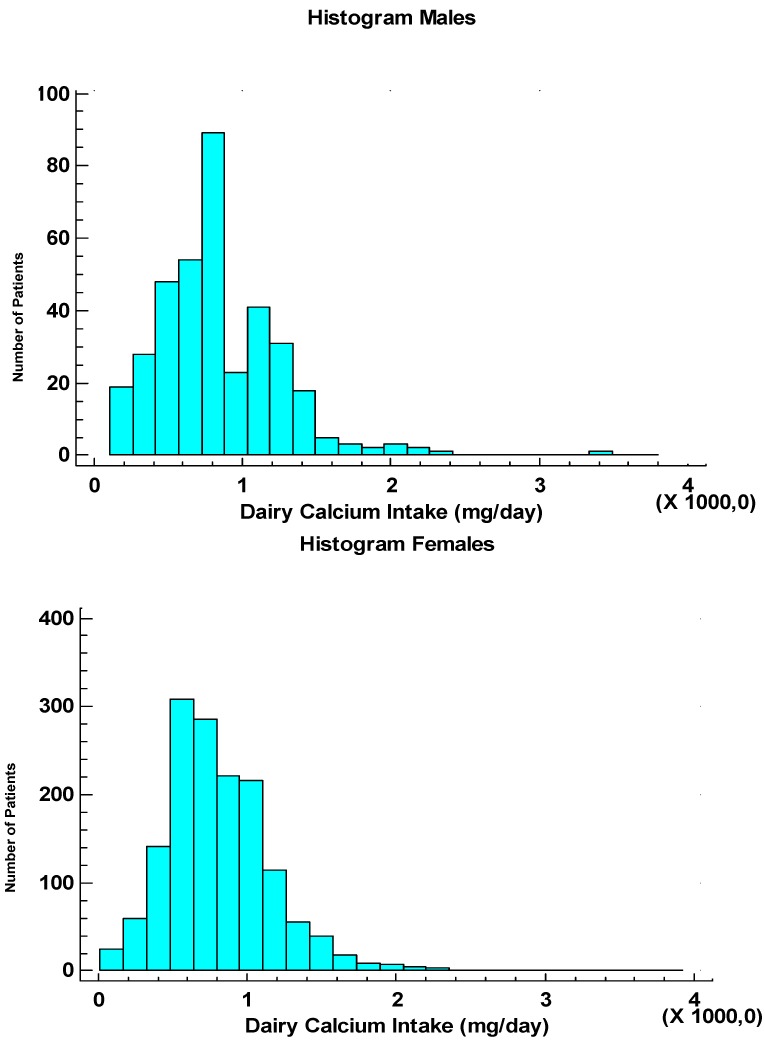
Distributions of daily calcium intake (mg/day) from dairy products in women and men.

From March 2008 to November 2011, we included 1526 women and 372 men of 50 years of age and older with a recent fracture. All patients were Caucasians. Of these patients, 1451 women and 349 men received a Dual Energy X-ray Absorptiometry (DXA: Hologic QDR 4500 C) of the total hip, femoral neck, and lumbar spine and had a consultation at the outpatient clinic with a specialized nurse practitioner. Relevant complete data were obtained from all 1898 patients who were originally included. The demographics are listed in Table 1 and Table 2. Calculated daily dairy calcium intakes in genders are graphically represented in Figure 1 and the distribution of genders into age decades is listed in Table 3.

**Table 1:** Demographic data of a Dutch cohort after a recent fracture.

Sex	Numbers	Age	% Osteoporosis (T-Score < −2.5 SD)	FRAX, % (for Major Fracture)	FRAX, % (for Hip Fracture)
Women	1526	66 (50–96)	13.6	11.0 (2.4–90.0)	2.6 (0.0–73.0)
Men	372	65 (50–90)	6.2	7.5 (1.6–41.0)	2.7 (0.1–37.0)

Fracture Assessment Tool (FRAX) was used to estimate FRAX risk scores for major osteoporotic fracture or hip fracture. Data were not normally distributed and are given as Median (Range).

**Table 2:** Variables of a Dutch cohort after a recent fracture.

Variables	Data	
**Sex**		
Women	0	
Men	1	
**Age Decades**		
50–59 years	1	
60–69 years	2	
70–79 years	3	
80–89 years	4	
90–100 years	5	
**Age at Menopause**	age (years), women only	
**Population Density**		
category 1	21–250 inhabitants/km^2^	
category 2	250–500 inhabitants/km^2^	
category 3	500–1000 inhabitants/km^2^	
category 4	1000–2500 inhabitants/km^2^	
category 5	2500–5967 inhabitants/km^2^	
**Dairy Calcium Intake**		
per serving milk	270 mg calcium	
per serving yoghourt	240 mg calcium	
per serving cheese	160 mg calcium	
**Currently Smoking**		
No	0	
Yes	1	
**Alcohol**		
≥3 servings per day	1	
≤2 servings per day	0	
**Current use of corticosteroids**		
No	0	
Yes	1	
**Rheumatoid arthritis**	
No	0
Yes	1
**Previous fracture**	
No	0
Yes	1
**Hip fracture mother**	
No	0
Yes	1
**Current use of calcium prescribed or as OTC**	Excluded
**Current use of any drug against osteoporosis ^#^**	Excluded
**Length**	cm
**Weight**	kg
**BMI**	Body Mass Index: Weight/(Length × Length)
**BMD**	Bone Mineral Density test, BMD-at-the-femoral neck (T-score)
**FRAX**	10-year probability of major osteoporotic fracture
**FRAX Hip**	10-year probability of hip fracture
**Secondary causes for osteoporosis known to the patient**	
No	0
Yes	1

Legends: OTC: Over-The-Counter available drugs not prescribed by a physician. ^#^ Any drug against osteoporosis, *i.e.*, bisphosphonates, estrogens or Selective Estrogen Receptor Modulators.

This study included more women than men, but genders were distributed equally over age decades, as is demonstrated in Table 2.

**Table 3:** Distribution of 1898 patients into five age decades (range 50–100 years).

Age (years)	Women W (*N* = 1526)	Men M (*N* = 372)	Ratio of Genders W/M
50–59	399 (26.1)	113 (30.4)	3.5
60–69	528 (34.6)	125 (33.6)	4.2
70–79	428 (28.0)	96 (25.8)	4.5
80–89	161 (10.6)	37 (10.0)	4.4
90–100	10 (0.7)	1 (0.2)	10.0.

Data for women and men per age decade are given in numbers of patients and percentage of patients (%).

Overall, we found no significant difference in calcium intake from dairy food products between women and men (median 790 (range 0–4360) and 790 (0–3435) mg/day, respectively. The dairy calcium intake was also expressed as numbers of servings per day, which were similar for each age decade except for those patients who were older than 80 years (Table 4). Numbers of servings did not differ between genders or between female and male cohorts compared for population density categories (Table 5).

**Table 4:** Number of daily servings of dairy products in females and males distributed into five age decades.

**Age (years)**	**Women (*N* = 1526)**	**Servings/Day**
50–59	399 (26.1)	3 (0–9)
60–69	528 (34.6)	3 (0–15)
70–79	428 (28.0)	3 (0–13)
80–89	161 (10.6)	3 (0–8)
90–100	10 (0.7)	3 (2–4)
**Age (years)**	**Men (*N* = 372)**	**Servings/Day**
50–59	113 (30.4)	3 (0–15)
60–69	125 (33.6)	3 (0–9)
70–79	96 (25.8)	3 (0–8)
80–89	37 (10.0)	3 (1–5)
90–100	1 (0.2)	1

Data for women and men per age decade are given in numbers of patients having completed the questionnaire and in percentage of patients (%). Data for number of servings are not normally distributed and are given as median (range).

**Table 5:** Number of daily servings of dairy products in women and men grouped by population density.

**Population Density Category (Inhabitants per km^2^)**	**Women (*N* = 1526)**	**Servings/day**
2 (250–500)	98 (6.4)	3 (1–10)
3 (500–1000)	5 (0.3)	4 (3–5)
4 (1000–2500)	565 (37.0)	3 (0–9)
5 (2500–5976)	858 (56.3)	3 (0–15)
**Population Density Category (Inhabitants per km^2^)**	**Men (*N* = 372)**	**Servings/day**
2 (250–500)	19 (5.1)	3 (1–6)
3 (500–1000)	2 (0.5)	4 (3–5)
4 (1000–2500)	156 (41.9)	3 (0–15)
5 (2500–5976)	195 (52.5)	3 (0–9)

Inhabitants per km^2^ per category was defined according to the Dutch National Atlas of Public Health [13]. Category 1 was not represented in the study region. Data for women and men per population density category are given in numbers of patients and in percentage of patients (%). Data for number of servings are not normally distributed and are given as median (range).

Most women and men were osteopenic and used 500–1000 mg calcium calculated from total dairy product intake per day. No significant differences in patients in both genders were found for dairy intake or for DXA T-scores: normal BMD, osteopenia or osteoporosis (Table 6).

**Table 6:** Stratified Calcium intakes from dairy products in women and men grouped by Dual Energy X-Ray Absorptiometry (DXA) T-scores.

**Dairy Calcium Intake (mg/day)**	**Women (*N* = 1451)**		
	**DXA T–Score (SD)**		
	≥−1	−1.0 to −2.5	≤−2.5
<500	42 (2.9)	141 (9.7)	49 (3.4)
500–1000	182 (12.5)	445 (30.7)	152 (10.5)
1000–1500	87 (6.0)	228 (15.7)	59 (4.1)
1500–2000	12 (0.8)	34 (2.3)	6 (0.4)
≥2000	4 (0.3)	7 (0.5)	3 (0.2)
**Dairy Calcium Intake (mg/day)**	**Men (*N* = 349)**		
	**DXA T–Score (SD)**		
	≥−1	−1.0 to −2.5	≤−2.5
<500	12 (3.4)	37 (10.6)	9 (2.6)
500–1000	53 (15.2)	118 (33.8)	14 (4.0)
1000–1500	21 (6.0)	62 (17.8)	6 (1.7)
1500–2000	2 (0.6)	9 (2.6)	0 (0.0)
≥2000	0 (0.0)	6 (1.7)	0 (0.0)

There were no significant differences between calcium intake from dairy per DXA T-score category, neither within gender nor between genders.

The median (range) FRAX 10-year probability for major osteoporotic fracture was significantly higher in women than in men (11.0% (2.4–90.0) and 7.5% (1.6–41.0), respectively, *p* < 0.05). Ten-year probability of hip fracture (median (range)) was not significantly different between genders (2.6% (0.0–73.0) in women and 2.7% (0.1–37.0) in men, respectively).

**Figure 2:**
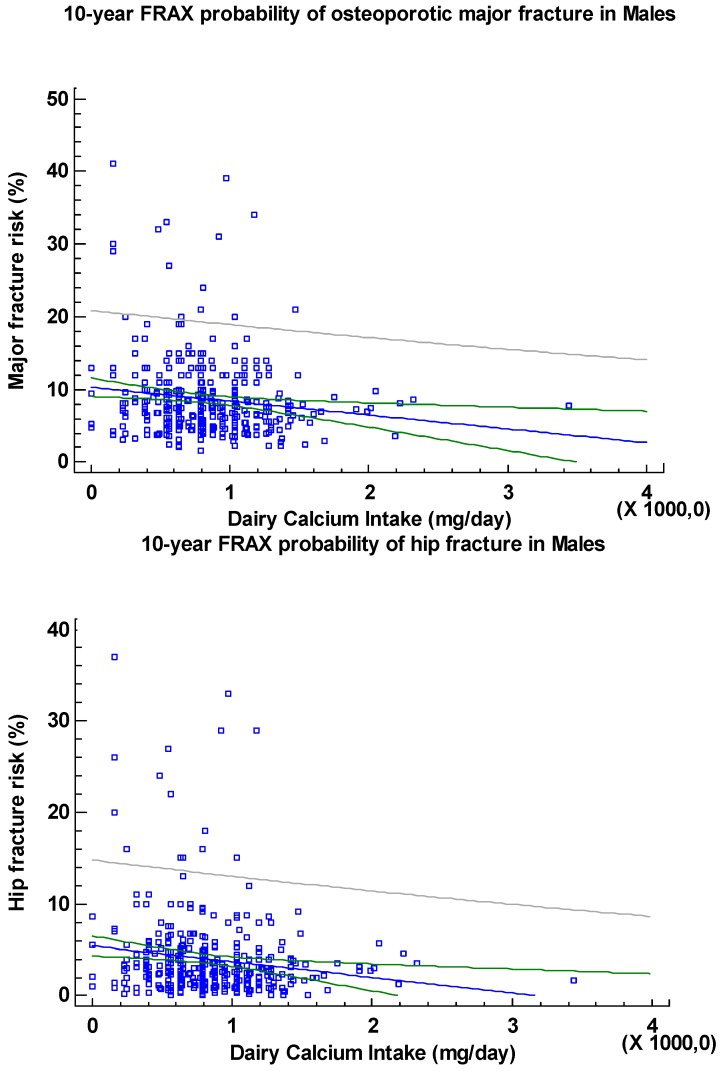
Relationship between daily dairy calcium intake and 10-year FRAX probability of osteoporotic major fracture or hip fracture in men.

In men (Figure 2), dairy calcium intake showed a weak but significant negative correlation with the 10-year FRAX probability of major fracture as well as of hip fracture (Rho: −0.13 and −0.14, respectively, *p* < 0.01), but no significant correlation with age, height, Body Mass Index (BMI), weight or BMD at the femoral neck. In females, we found no significant correlation between dairy calcium intake and the variables mentioned or with “age at menopause”.

Logistic regression analysis disclosed “maternal hip fracture” as a dependent variable. This was not found for any of the other variables like age, BMI, age at menopause (women only), population density, dairy calcium intake, more than one fracture prior to analysis, currently smoking, current glucocorticoids use, rheumatoid arthritis, secondary causes for osteoporosis known to the patient, alcohol use (either ≤2 or ≥3 servings), BMD at the femoral neck, 10-year probability of osteoporotic major fracture, and 10-year probability of hip fracture. In women, logistic regression showed a weak relationship between “maternal hip fracture” and the independent variables (factors) BMD-at-the-femoral neck, 10-year probability of osteoporotic major fracture, 10-year probability of hip fracture, BMI, height, weight, alcohol use = 0, currently smoking = No. In men, logistic regression showed a strong relationship between “maternal hip fracture” and the independent variables BMD at the femoral neck, 10-year probability of osteoporotic major fracture, 10-year probability of hip fracture, BMI, and Prednisone = 0.

### Comparison between the Current Dutch Cohort and a Cohort from the U.S.

The percentage of patients fulfilling the recommended total calcium intake of >1100 mg/day (≤69 years) or >1200 mg/day (>70 years) depends on the total amount of calcium in Dutch basic nutrition. In this study, this would mean that focusing on dairy intake alone, only 11.3% of the women and 14.2% of the men comply with Dutch recommendation. However, assuming that Dutch basic non-dairy nutrition provides 42% of the total daily calcium intake (P50 = 450 mg) [6], our findings mean that 60.5% of women and 59.1% of men comply with Dutch recommendations for nutritional calcium intake. U.S. data from the group of Fulgoni [2] showed a mean calcium intake of 674 ± 6 mg/day from dairy products for both women and men older than 50 years; 15.2% of both women and men complied with U.S. recommendations for nutritional calcium intake. Moreover, U.S. non-dairy nutrition was estimated to contain 250–475 mg calcium, and complemented with 2.93 dairy servings this would imply that 53.2% of this group complied with U.S. recommendations for calcium from daily nutrition [3].

## 4. Discussion

The current study shows that calcium intake from dairy products in a Dutch cohort of Caucasians with a recent fracture was well below the Dutch RDI of 1100 mg for individuals between 50 and 70 years and of 1200 mg for older people. There were no differences in dairy calcium intake between age decades, genders or population density categories. In contrast to women, in men there was a weak but significant negative correlation between dairy calcium intake and 10-year probability of major fracture and hip fracture. Importantly, the present study showed that prevalence of “maternal hip fracture” was a factor for current fracture risk, both in women and men.

In spite of the fact that the estimated calcium intake is lower in most European countries than in the U.S. [2], there were no significant differences between our cohort of fracture patients and a comparable non-fracture cohort from the U.S. in terms of dairy calcium intake (expressed as the consumed amount of calcium or as daily number of servings).

The current study demonstrates that only 11.3% of women and 14.2% of men comply with Dutch recommendations for daily calcium intake from dairy products. However, a different picture emerges after adding 450 mg calcium from basic nutrition [4,6]. Nevertheless, even in this scenario, 39.1% of women and 37.4% of men still do not comply with Dutch recommendations (*i.e.*, consume less calcium than recommended). We emphasize that our choice of 450 mg calcium from non-dairy nutrition is based on previously published data by the Dutch National Institute for Public Health and the Environment (RIVM) [6]. These estimates are clearly higher compared to those made by Heaney [14] and documented in the Clinician’s Guide to Prevention and Treatment of Osteoporosis by the National Osteoporosis Foundation (NOF), *i.e.*, 250 mg per day [15].

Whether this non-compliance with Dutch recommendations should be considered a health risk is debatable, since there is no uniformity amongst countries in the recommendation of calcium intake from dairy products or supplements. The Dutch daily recommendations for adults are similar to those in the U.S., *i.e.*, for men 50–70 years: 1000 mg, for women >50 years and men >71:1200 mg calcium per day (NOF Clinician’s guide). For Australia and New Zealand, recommendations are 1300 mg, for the UK 700 mg and for Scandinavian countries 800 mg. The exact amount of calcium intake optimal for skeletal health in adults remains an unresolved issue [16,17,18,19,20]. For example, daily calcium intake in Asian and African countries is about a third of that in Western countries [21], and in Japan it is no more than 400–500 mg/day [2].

The current study found weak but significant negative correlations between dairy calcium intake and 10-year probability of major fracture and 10-year probability of hip fracture in the male cohort. This implies that males with the lowest calcium intake are at highest risk for fracture.

These data agree with the age-adjusted incidence rates of hip fractures around the world, which show a negative association with calcium intake by country and hip fracture rates [17,22,23,24]. However, several other cohort studies in middle-aged and older Caucasian women and men found no proof of beneficial effects of dairy in reducing hip fractures [21,22,25,26]. Finally, a recent study by Feskanich *et al.* has shown that the highest hip fracture risk in men appears to be in those individuals who consumed most dairy products during their teenage years [27].

The different outcomes of studies with regard to effects of dairy consumption and osteoporotic fractures are confusing, but they may possibly be explained by dual mode effects of dairy consumption and the incidence of osteoporotic fractures. This phenomenon has previously been shown in the prospective longitudinal mammography cohort from Sweden analyzing the cumulative intake of dietary calcium study amongst 61.433 participants [21]. Primary outcome measures were incident fractures of any type and hip fracture (6% during 19 years of follow-up). From this large amount of data it appeared that the risk of low calcium intake in the lowest quintile was associated with an increased fracture risk (in all categories, as well as hip fractures). Oppositely, in the highest quintile of calcium intake, there was an increased risk but only for hip fractures. Moreover, the investigators showed a multivariable adjusted spline curve for the relation between cumulative average intake of dietary calcium and time to first hip fracture, suggesting a protective effect of dairy calcium intake amounting to 800 mg per day, whereas a fracture-promoting effect was found for intakes exceeding 800 mg of dietary calcium. In other words, it seems that the beneficial effects of dairy calcium intake followed a U-shaped pattern with calcium dose [21].

The variability of consumption of dairy products around the globe can be explained both by historical/cultural food aspects and biological variations, such as differences in intestinal lactase expression in populations. Lactase expression at infancy is universal, but in most mammals, its activity declines after the age of weaning. Lactase activity in humans, however, is variable. Although most populations of the world have a low prevalence of lactase persistence, Northern European populations tend to have a high preservation of intestinal lactase expression, while it is lowest among Asians [24]. Combined databases of non-cohort studies have shown that lactose intolerance and malabsorption are clearly less prevalent among individuals of Northern European descent than in African American, Hispanic, Asian, and American Indian populations [28].

While lactose intolerance can be a barrier to milk consumption, it has been shown that at least 240 mL of whole or skimmed milk per day (containing approximately 37.5 g lactose) may be consumed by lactose maldigesters without experiencing adverse symptoms, especially if amounts are divided into smaller doses taken throughout the day [29,30]. Most individuals with lactose intolerance can tolerate up to 12 g of lactose in a single dose, though symptoms will become more prominent at doses above 12 g and appreciably so after 25 g of lactose; 50 g will induce symptoms in the vast majority of lactose intolerant individuals [28].

While U.S. authorities support supplemental milk feeding programs [29] it is in this respect remarkable to note that three servings of dairy products a day are found to be uniform among adult populations of North-Western European descent, regardless of age, gender or population density, the latter being true at least for the Netherlands. Recommending more than three daily dairy servings to protect adult bones in North-Western European populations does not seem to have any scientific grounds.

An important finding of the present study is that the prevalence of maternal hip fracture is an outcome variable for current fracture risks, both in men and women. These findings are in line with the publication and data of Lalmohamed [11] who investigated and calibrated the Dutch version of the FRAX algorithm. Our calculations were based on logistic regression models applying numeric as well as dichotomized variables (*i.e.*, smoking, alcohol use, prednisone use, rheumatoid arthritis) and applying the FRAX algorithm in a fracture cohort. Our data indicate that genetics may play a more prominent role in osteoporotic fractures, independent of gender. Moreover, when calibrating FRAX for our Dutch cohort, we also found that maternal hip fracture accounted for the greatest increase in 10-year fracture probability.

A possible limitation of our study is that the Dutch data were obtained from patients who sustained a fracture, while the U.S. data were obtained from healthy individuals. It may be argued that a recent fracture may bias memorized daily dairy intakes. Another limitation of our study might be the fact that we did not focus on the vitamin D levels. These assays were not systematically performed in the included patients.

## 5. Conclusions

We have shown that Dutch fracture patients are used to consuming, on average, three servings of dairy products per day, containing approximately 750 mg of calcium, plus an estimated 450 mg extra calcium from basic nutrition. There were no differences in daily dairy intake between age cohorts, genders or population densities. While daily dairy calcium intake of Dutch fracture patients was well below the recommended dietary intake, it is comparable to healthy U.S. cohorts composed of mainly Caucasians. This questions the recommendations to use additional amounts of dairy products to preserve adult skeletal health, particularly when sufficient additional calcium is derived from adequate non-dairy basic nutrition.
